# Symptoms associated with adverse dengue fever prognoses at the time of reporting in the 2015 dengue outbreak in Taiwan

**DOI:** 10.1371/journal.pntd.0006091

**Published:** 2017-12-06

**Authors:** Chun-Yin Yeh, Po-Lin Chen, Kun-Ta Chuang, Yu-Chen Shu, Yu-Wen Chien, Guey Chuen Perng, Wen-Chien Ko, Nai-Ying Ko

**Affiliations:** 1 Institute of Allied Health Sciences, College of Medicine, National Cheng Kung University, Tainan, Taiwan; 2 Department of Medicine, College of Medicine, National Cheng Kung University, Tainan, Taiwan; 3 Department of Computer Science and Information Engineering, National Cheng Kung University, Tainan, Taiwan; 4 Department of Mathematics, National Cheng Kung University, Tainan, Taiwan; 5 Department of Public Health, College of Medicine, National Cheng Kung University, Tainan, Taiwan; 6 Department of Occupational and Environmental Medicine, National Cheng Kung University Hospital, Tainan, Taiwan; 7 Department of Microbiology and Immunology, College of Medicine, National Cheng Kung University, Tainan, Taiwan; 8 Department of Nursing, College of Medicine, National Cheng Kung University and Hospital, Tainan, Taiwan; Baylor College of Medicine, UNITED STATES

## Abstract

**Background:**

Tainan experienced the most severe dengue epidemic in Taiwan in 2015. This study investigates the association between the signs and symptoms at the time of reporting with the adverse dengue prognoses.

**Methods:**

A descriptive study was conducted using secondary data from the Dengue Disease Reporting System in Tainan, Taiwan, between January 1 and December 31, 2015. A multivariate stepwise logistic regression was used to identify the risk factors for the adverse prognoses: ICU admissions and mortality.

**Results:**

There were 22,777 laboratory-confirmed reported cases (mean age 45.6 ± 21.2 years), of which 3.7% were admitted to intensive care units (ICU), and 0.8% were fatal. The most common symptoms were fever (92.8%), myalgia (26.6%), and headache (22.4%). The prevalence of respiratory distress, altered consciousness, shock, bleeding, and thrombocytopenia increased with age. The multivariate analysis indicated that being in 65–89 years old age group [Adjusted Odds Ratio (aOR):4.95], or the 90 years old and above age group (aOR: 9.06), and presenting with shock (aOR: 8.90) and respiratory distress (aOR: 5.31) were significantly associated with the risk of ICU admission. While old age (aOR: 1.11), respiratory distress (aOR: 9.66), altered consciousness (aOR: 7.06), and thrombocytopenia (aOR: 2.55) were significantly associated with the risk of mortality.

**Conclusions:**

Dengue patients older than 65 and those with severe and non-specific signs and symptoms at the time of reporting were at a higher risk of ICU admission and mortality. First-line healthcare providers need to be aware of the varied presentations between the different age groups to allow early diagnosis and in-time management, which would prevent ICU admissions and fatalities in dengue patients.

## Author summary

Clinical presentations of dengue fever (DF) are diverse and non-specific and often have unpredictable progression and outcomes. The patients in the 2015 dengue epidemics in Taiwan were predominantly much older than in other countries in Southeast Asia. However, limited data are available in Taiwan on dengue patients with adverse prognoses who were admitted to ICU or on those with fatal complications. All suspected dengue cases were reported to the Dengue Disease Reporting System in Taiwan. We examined patients who had laboratory-confirmed dengue in Tainan during the 2015 dengue outbreak and analyzed secondary data from the reporting system. Of these patients, 3.7% were admitted to ICU, and 0.8% died. Patients who received adverse prognoses were generally of older age, had a critical presentation at the time of reporting, and experienced a rapid progress of the disease, highlighting that people with severe and non-specific signs and symptoms at the time of reporting were at a higher risk of ICU admission and mortality.

## Introduction

Dengue is a mosquito-borne viral disease that has become a major public health problem owing to its wide geographical extension, high incidence, and disease severity [1, 2]. Southeast Asia and the Western Pacific are most seriously affected by dengue, with 75% of the current globally reported outbreaks [1]. Taiwan has experienced three dengue outbreaks in recent years: the first in Penghu County in 2011 (prevalence rate 101 per 100 000 population), the second in Kaohsiung City in 2014 (prevalence rate 540 per 100 000 population), and the most recent outbreak in Tainan in 2015 (prevalence rate 1,208 per 100 000 population) [3]. The 2015 Tainan dengue outbreak, caused by dengue serotype 2, was the most severe epidemic in Tainan’s history [4], with 2.99–14.9% of dengue patients being admitted to the intensive care unit (ICU) [5, 6] and 0.52% dying [7]. Patients aged over 70 years had the highest prevalence rate [3], which was in contrast to the predominantly younger dengue patients in other Southeast Asian countries [8–14].

Dengue presentations are diverse and non-specific and often have unpredictable clinical progression and outcomes [1]. While most patients recover from DF, approximately 0.3–14.9% develop severe manifestations that result in ICU admission [5, 6, 13, 15], and 1–5% die without early recognition and proper treatment [1]. Timely access to proper treatment for dengue patients by primary healthcare professionals not only reduces the number of unnecessary hospital admissions but also lowers fatality rates below 1% [1]. The sensitivity of the World Health Organization (WHO) 2009 classification systems was 52% in differentiating patients who required ICU admissions at first presentation [13]. However, according to the WHO 2009 classification systems, as sensitivity decreases with age, it is difficult to differentiate dengue from other clinically febrile diseases in older patients [16]. Therefore, a better understanding of the signs and symptoms at the time of reporting associated with poor prognoses may assist first-line healthcare providers to focus on patients who at higher risk and enable timely treatment, especially for the aging dengue population.

Only one case-control study in Singapore has reported clinical factors associated with ICU admission in dengue patients; 50–59 year age group, diabetes, the WHO 2009 classification of dengue severity, hematocrit change greater or equal to 20% concurrent with platelets less than 50,000/μl, hypoproteinemia, hypotension, and severe organ involvement [13, 17]. However, there have been several studies that have identified the risk factors associated with dengue mortality: old age [18–21], being female [18], and presenting with symptoms such as nausea and vomiting [18], bleeding [18, 22], gastrointestinal bleeding [20], hematuria [20], thrombocytopenia [20], leukocytosis [23], altered mental status [18, 22], plasma leakage [18, 21], cavity effusions [19], tachycardia [24], and shock [18, 22]. Most of these studies were limited, however, because of small sample sizes [13, 23] and single hospital studies [22, 24], or were focused primarily on severe dengue patients [19–21]. A study in Malaysia examined the national registry data of 43,347 dengue patients in 2013; however, the dengue diagnosis was verified using WHO 1997 criteria [18], which had a dengue severity sensitivity and specificity lower than the WHO 2009 [25, 26], and only 30.2% of patients with DF were serologically confirmed [18]. In addition, the mean age of patients with DF in Malaysia was 30 [18], which was much younger than the majority of the dengue population in Taiwan [3]. There is a lack of information about the signs and symptoms at the time of reporting associated with ICU admissions and mortality in DF patients in aging societies such as Taiwan. This study seeks to describe the signs and symptoms at the time of reporting in dengue patients across different age groups and identify the signs and symptoms associated with ICU admission and mortality in 2015 dengue patients in Tainan, Taiwan.

## Methods

### Study design and data source

Using registry data from the Dengue Disease Reporting System from January 1 and December 31, 2015, the retrospective cohort study included all 2015 dengue patients in Tainan, Taiwan. In Taiwan, according to the Law on the Control of Communicable Diseases, all suspected dengue cases must be reported to the Health Department at the Tainan and Taiwan Centers of Disease Control (Taiwan CDC) within 24 hours [4]. The dengue-infected cases were diagnosed from laboratory results based on the following criteria; a reverse-transcription polymerase chain reaction (RT-PCR), an evaluation of the anti-dengue virus IgM and IgG, and dengue viral isolation from serum or tissue [27]. The specimens were confirmed by the approved laboratories at Taiwan CDC after which final confirmations were performed [28]. The decision to admit a DF patient to ICU or not was judged by the treating physician and was not documented by the reporting system. However, Taiwan national guidelines on dengue fever management are available and clinicians could treat the critical dengue patients accordingly [28].

This study was exempt from a full review by the Institutional Review Board of National Cheng Kung University Hospital (no. A-ER-104-386) as the database consisted of de-identified data and a confidentiality agreement with Tainan City Government was signed by all researchers using the dataset. The data included age, gender, the status of the patient at the time of reporting, the region of patients who lived when being reported, the levels of hospitals/clinics reporting dengue cases, dates for the dengue onset, reporting dates to Taiwan CDC, dates of the dengue confirmations by the Taiwan CDC, dates of the mortalities, the signs and symptoms at the time of reporting, the presence or absence of severe symptoms, information about ICU admission, and the mortality reported by the physicians. The reporting data were updated and confirmed by the medical officers at Taiwan CDC. Patients without complete data were excluded from analysis.

### Definitions

The primary outcomes included ICU admission and mortality. ICU admission was defined as the ICU admission details in dengue-infected people on the latest version before the data analysis, mortality was defined as the death registration of the confirmed dengue patients, and accuracy was verified by cross matching the discharge data from the three major hospitals in Tainan, which between them had seen 9,816 dengue patients.

The independent variable was the signs and symptoms in dengue-infected people at the time of reporting. The signs and symptoms reported by physicians were verified by the Tainan Health Department and were classified into thirty-seven categories based on the WHO (2009) classification system [1, 4]. To avoid misclassification, the classification system was further validated by a programmer, an epidemiologist, and an infectious disease clinician (S1 Table). The other variables included age: <15 years, 15–39, 40–64, 65–89, and over 90 years, gender, the status of patients at the time of reporting, the region of patients who lived when being reported, the levels of hospitals/clinics reporting dengue cases, dates for dengue illness onset, reporting dates to Taiwan CDC, dates of dengue confirmation by Taiwan CDC, dates of ICU admission and death, the presence or absence of severe symptoms.

### Statistical analysis

The differences between the groups (different age group, ICU and non-ICU, survivors and non-survivors) were examined using t-tests or median test for the continuous variables, and an *χ*^2^ test for the categorical variables. Patients who were admitted to ICU or died during reporting were excluded from multivariate analysis, and then the univariate logistic regression was performed using the demographic variables (age and gender), the levels of hospitals/clinics reporting dengue cases, and the signs and symptoms as the independent variables and ICU admission and mortality as the dependent variables. A p value less than 0.05 was considered potentially significant and was further analyzed with multivariate stepwise logistic regression using the Allen-Cady modified backward selection method to identify the significant demographic variables and the signs and symptoms at the time of reporting that led to ICU admission and mortality [29]. An odds ratio (OR) and 95% confidence interval (CI) were considered significant at a p value ≤ 0.05. IBM SPSS Statistics 19 software was used for the analyses in this study.

## Results

### Study population

A total of 22,777 laboratory-confirmed dengue patients in Tainan were included in this study. Of these, 22,737 (99.8%) patients reported the presence of at least one sign and symptom at the time of reporting to Taiwan CDC. Of the confirmed dengue patients, 3.3% (396/11,922) had severe symptoms, 3.7% (337/9197) were admitted to the ICU. Among them, 1.3% (131/9816) died at the three major hospitals in Tainan, and 0.8% (189/22777) died during the 2015 dengue outbreak in Tainan. Table 1 describes the characteristics of the confirmed cases in the 2015 dengue outbreak in Tainan. The mean age was 45.6 years (standard deviations [SD] = 21.2), 50.4% were female, 44.7% were reported at the local clinics, and 30.8% were reported at the emergency departments of either regional hospitals or medical centers. The majority of the reported cases (74.1%) lived in the urban region of Tainan, and 39.5% were reported by the regional hospitals. The median days between illness onset and reporting to Taiwan CDC was 1 day (interquartile range [IQR], 1–3), and 2 days between illness onset and dengue confirmation by Taiwan CDC (IQR, 1–4). The median days between dengue confirmation and ICU admission was 0 days (IQR, -1–2), and 3 days (IQR, 1–13) between dengue confirmation and death. Patients admitted to the ICU admission were predominantly male, older, and more likely to be the residents in the rural region and being reported by medical centers. Of note, they were apt to have severe symptoms, and had longer days between illness confirmation and death. Patients who died were older, more likely to be reported at emergency departments and medical centers. Similar to patients admitted to ICU, they were more likely to have severe symptoms, but had shorter days between illness onset and dengue reporting or confirmation (Table 1).

**Table 1:** Characteristics of the 22,777 patients with dengue in Tainan.

Variables	Total	Non-ICU	ICU	p	Survivors	Non-survivors	p
	N = 22,777	N = 8,860	n = 337		N = 22,588	n = 189	
**Age**	45.6 ± 21.2	46.3 ± 21.0	70.5 ± 15.6	0.0001	45.4 ± 21.04	74.94 ± 12.1	0.0001
<15	1788 (7.9)	640 (7.2)	11 (3.3)		1788 (7.9)	0 (0.0)	
15–39	7426 (32.6)	2784 (31.4)	6 (1.8)		7422 (32.9)	4 (2.1)	
40–64	8809 (38.7)	3580 (40.4)	44 (13.1)		8788 (38.9)	21 (11.1)	
65–89	4678 (20.5)	1833 (20.7)	268 (79.5)		4529 (20.1)	149 (78.8)	
90+	76 (0.3)	23 (0.3)	8 (2.4)		61 (0.3)	15 (7.9)	
**Gender**				0.045			0.408
Female	11469 (50.4)	4556 (51.4)	154 (45.7)		11380 (50.4)	89 (47.1)	
Male	11308 (49.6)	4304 (48.6)	183 (54.3)		11208 (49.6)	100 (52.9)	
**The status of patients when being reported (n = 22416)**				0.0001			0.0001
General ward treatment	1657 (7.4)	913 (10.3)	59 (17.5)		1625 (7.3)	32 (16.9)	
ICU treatment	110 (0.5)	0 (0.0)	110 (32.6)		78 (0.4)	32 (16.9)	
Death	10 (0.0)	6 (0.1)	1 (0.3)		0 (0.0)	10 (5.3)	
Clinics	10009 (44.7)	4083 (46.1)	47 (13.9)		9988 (44.9)	21 (11.1)	
Emergency department	6899 (30.8)	2718 (30.7)	96 (28.5)		6816 (30.7)	83 (43.9)	
Other (Transfer to another hospital/Discharged)	3731 (16.6)	1140 (12.9)	24 (7.1)		3720 (16.7)	11 (5.8)	
**The region of patients who lived when being reported**				0.0001			0.174
Urban	16874 (74.1)	7267 (82.0)	215 (63.8)		16733 (74.1)	141 (74.6)	
Rural	5626 (24.7)	1434 (16.2)	118 (35.0)		5583 (24.7)	43 (22.8)	
Other	277 (1.2)	159 (1.8)	4 (1.2)		272 (1.2)	5 (2.6)	
**The levels of hospitals/clinics reporting dengue cases**				0.0001			0.0001
Medical center	8436 (37.0)	968 (10.9)	159 (47.2)		8330 (36.9)	106 (56.1)	
Regional hospital	8987 (39.5)	6165 (69.6)	168 (49.9)		8915 (39.5)	75 (38.1)	
District hospital	422 (1.9)	165 (1.9)	1 (0.3)		420 (1.9)	2 (1.1)	
Clinics	4163 (18.3)	1292 (14.6)	7 (2.1)		4157 (18.4)	6 93.2)	
Other	769 (3.4)	270 (3.0)	2 (0.6)		766 (3.4)	3 (1.6)	
**Days between illness onset and reporting**	1 (1–3)	1 (1–3)	1 (1–3)	0.637	1 (1–3)	1 (1–2)	0.001
**Days between illness onset and confirmation**	2 (1–4)	2 (1–4)	2 (1–4)	0.698	2 (1–4)	2 (1–3)	0.041
**Days between illness confirmation and ICU admission**	0 (-1–2)	—	0 (-1–2)	—	0 (-1–2)	0 (-1–2)	0.700
**Days between illness confirmation and death**	3 (1–13)	2 (-1–4.5)	4 (1.250–12)	0.047	—	3 (1–13)	—
**Severe symptoms (n = 11922)**	396 (3.3)	62 (0.8)	262 (77.7)	0.0001	272 (2.3)	124 (66.7)	0.0001
**Presence of at least one symptom**	22737 (99.8)	8848 (99.9)	337 (100)	1.000	22549 (99.8)	188 (99.5)	0.244

Data are expressed as case number (%), mean ± standard deviation, median (interquartile range).

### Signs and symptoms at the time of reporting in the study population

**Fig 1:**
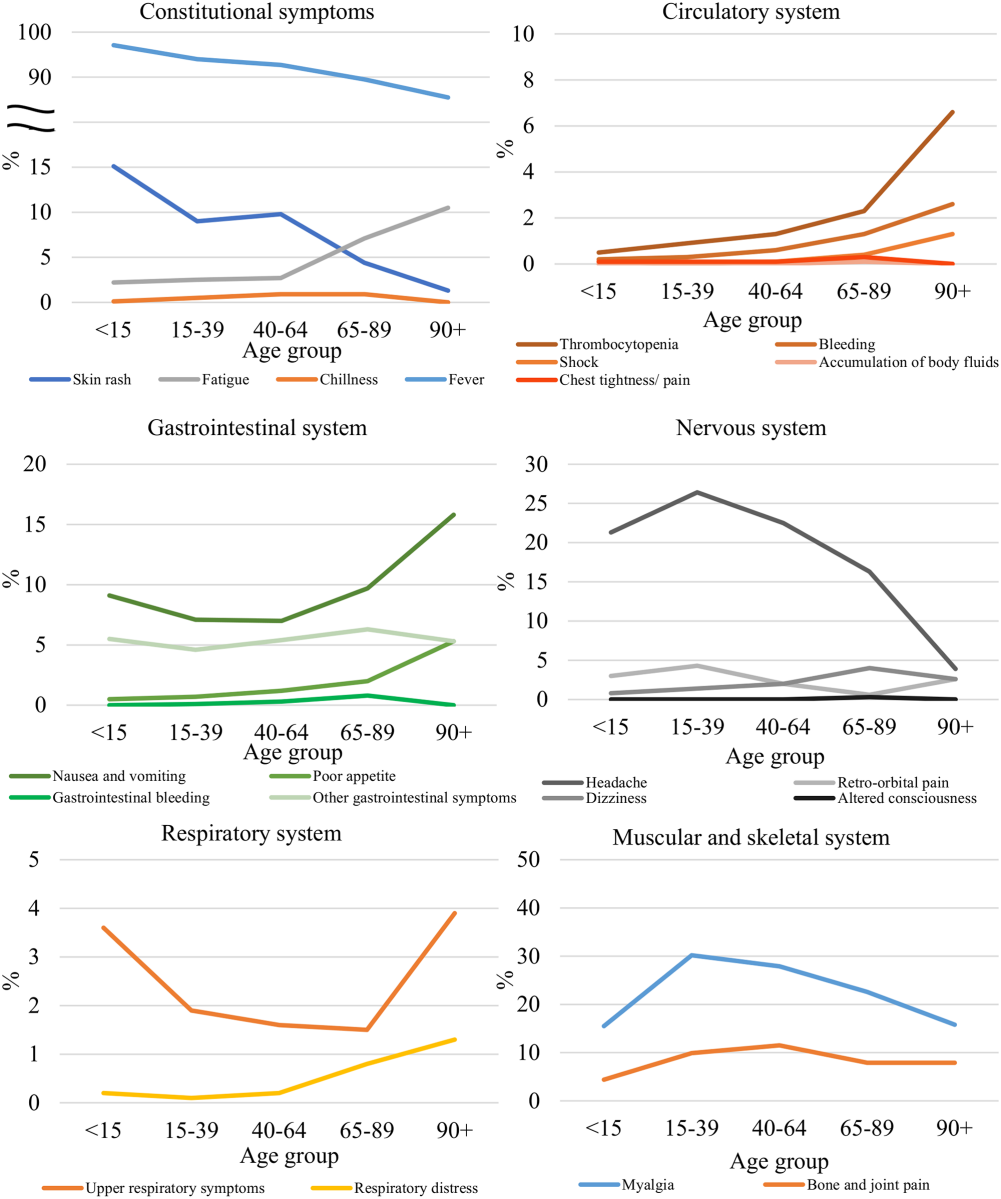
Distribution of the signs and symptoms by organ systems at the time of reporting in dengue patients in different age groups.

Table 2 shows a summary of the signs and symptoms at the time of reporting. Fever (92.8%), myalgia (26.6%), and headache (22.4%) were the most common symptoms. Fig 1 shows the distribution of the signs and symptoms at the time of reporting in dengue patients in the different age groups. In patients aged older than 65, the proportion with fever, myalgia, headaches, and skin rashes was significantly lower with an increase in age (p = 0.0001). The following signs and symptoms were more prevalent with an increase in age: nausea and vomiting, poor appetite, fatigue, thrombocytopenia, bleeding, respiratory distress, altered consciousness, shock, gastrointestinal symptoms, and chest tightness/pain.

**Table 2:** Signs and symptoms at the time of reporting in the 22,777 dengue patients in Tainan.

Symptoms	Total	Non-ICU	ICU	p	Survivors	Non-survivors	p
	N	n	n		n	n	
**Fever**	21135 (92.8)	8301 (93.7)	282 (83.7)	0.0001	20972 (92.8)	163 (86.2)	0.0001
**Myalgia**	6048 (26.6)	2202 (24.9)	56 (16.6)	0.001	6031 (26.7)	17 (9.0)	0.0001
**Headache**	5093 (22.4)	2411 (27.2)	34 (10.1)	0.0001	5075 (22.5)	18 (9.5)	0.0001
**Bone and joint pain**	2197 (9.7)	935 (10.6)	11 (3.3)	0.0001	2191 (9.7)	6 (3.2)	0.002
**Skin rash**	2005 (8.8)	811 (9.2)	9 (2.7)	0.0001	1998 (8.8)	7 (3.7)	0.013
**Nausea and vomiting**	1780 (7.8)	818 (9.2)	36 (10.7)	0.368	1767 (7.8)	13 (6.9)	0.630
**Gastrointestinal symptoms**	1217 (5.4)	441 (5.0)	27 (8.0)	0.013	1199 (5.3)	18 (9.5)	0.010
**Fatigue**	798 (3.5)	360 (4.1)	40 (11.9)	0.0001	784 (3.5)	14 (7.4)	0.003
**Leukopenia**	795 (3.5)	383 (4.3)	21 (6.2)	0.093	787 (3.5)	8 (4.2)	0.577
**Retro-orbital pain**	586 (2.6)	238 (2.7)	1 (0.3)	0.007	586 (2.6)	0 (0.0)	0.025
**Dizziness**	484 (2.1)	207 (2.3)	16 (4.7)	0.005	477 (2.1)	7 (3.7)	0.131
**Upper respiratory symptoms**	423 (1.9)	191 (2.2)	3 (0.9)	0.113	419 (1.9)	4 (2.1)	0.791
**Thrombocytopenia**	302 (1.3)	204 (2.3)	17 (5.0)	0.001	292 (1.3)	10 (5.3)	0.0001
**Poor appetite**	264 (1.2)	151 (1.7)	3 (0.9)	0.253	263 (1.2)	1 (0.5)	0.416
**Chillness**	156 (0.7)	72 (0.8)	2 (0.6)	0.658	156 (0.7)	0 (0.0)	0.643
**Bleeding**	144 (0.6)	53 (0.6)	17 (5.0)	0.0001	138 (0.6)	6 (3.2)	0.0001
**Tourniquet test positive**	109 (0.5)	31 (0.3)	1 (0.3)	0.871	108 (0.5)	1 (0.5)	0.919
**Gastrointestinal bleeding**	70 (0.3)	25 (0.3)	10 (3.0)	0.000	68 (0.3)	2 (1.1)	0.061
**Respiratory distress**	62 (0.3)	17 (0.2)	12 (3.6)	0.000	49 (0.2)	13 (6.9)	0.000
**Thirsty**	49 (0.2)	21 (0.2)	0 (0.0)	1.000	49 (0.2)	0 (0.0)	1.000
**Itching**	38 (0.2)	22 (0.2)	0 (0.0)	1.000	38 (0.2)	0 (0.0)	1.000
**Chest tightness/ pain**	34 (0.1)	14 (0.2)	1 (0.3)	0.536	32 (0.1)	2 (1.1)	0.001
**Shock**	27 (0.1)	4 (0.0)	12 (3.6)	0.0001	24 (0.1)	3 (1.6)	0.0001
**Abnormal liver function**	22 (0.1)	5 (0.1)	2 (0.6)	0.0001	22 (0.1)	0 (0.0)	1.000
**Urinary tract injury**	21 (0.1)	13 (0.1)	1 (0.3)	0.488	21 (0.1)	0 (0.0)	1.000
**Altered consciousness**	20 (0.1)	4 (0.0)	7 (2.1)	0.0001	15 (0.1)	5 (2.6)	0.0001
**Cold sweating**	9 (0.0)	4 (0.0)	0 (0.0)	1.000	9 (0.0)	0 (0.0)	1.000
**Accumulation of body fluids**	9 (0.0)	5 (0.1)	1 (0.3)	0.09	9 (0.0)	0 (0.0)	1.000
**Abnormal heart rhythm**	9 (0.0)	2 (0.0)	0 (0.0)	1.000	8 (0.0)	1 (0.5)	0.001
**Epilepsy**	4 (0.0)	1 (0.0)	0 (0.0)	1.000	4 (0.0)	0 (0.0)	1.000
**Severe bleeding**	2 (0.0)	0 (0.0)	0 (0.0)	---	0 (0.0)	2 (1.1)	0.0001
**Enlargement of lymph nodes**	1 (0.0)	0 (0.0)	0 (0.0)	---	1 (0.0)	0 (0.0)	1.000
**Mouth ulcers**	1 (0.0)	1 (0.0)	0 (0.0)	1.000	1 (0.0)	0 (0.0)	1.000
**Hepatosplenomegaly**	1 (0.0)	1 (0.0)	0 (0.0)	1.000	0 (0.0)	1 (0.5)	0.008
**Pneumonia on X-rays**	1 (0.0)	0 (0.0)	1 (0.3)	0.037	1 (0.0)	0 (0.0)	1.000
**Secondary infection**	1 (0.0)	0 (0.0)	0 (0.0)	---	1 (0.0)	0 (0.0)	1.000
**Muscle symptoms**	1 (0.0)	0 (0.0)	0 (0.0)	---	1 (0.0)	0 (0.0)	1.000

Data are expressed as case numbers (%).

Patients who presented with fever, headache, or myalgia at the time of reporting had shorter days between illness onset and reporting than those without these symptoms (fever: 2.1 ± 2.1 vs. 2.7 ± 2.9, p = 0.0001; headache: 1.9 ± 1.8 vs. 2.2 ± 2.2, p = 0.0001; myalgia: 2.0 ± 1.8 vs. 2.2 ± 2.2, p = 0.0001).

Patients who died and patients admitted to the ICU were significantly less likely to have fever, myalgia, headaches, bone and joint pain, skin rashes, and retro-orbital pain at the time of reporting compared to the survivors and non-ICU admitted patients. Patients who were admitted to the ICU had higher proportions of the following signs and symptoms at the time of reporting: gastrointestinal symptoms, fatigue, dizziness, thrombocytopenia, bleeding, gastrointestinal bleeding, respiratory distress, shock, abnormal liver function, altered consciousness, and pneumonia on X-rays. The patients who died had higher proportions of following signs and symptoms at the time of reporting: gastrointestinal symptoms, fatigue, thrombocytopenia, bleeding, respiratory distress, chest tightness/pain, shock, altered consciousness, abnormal heart rhythm, severe bleeding, and hepatosplenomegaly (Table 2).

### Signs and symptoms at the time of reporting associated with ICU admission or mortality

Table 3 shows the multivariable analysis of the factors associated with ICU admission in the 2015 dengue outbreak in Tainan (n = 9, 087). Of dengue-infected people, 10 patients died and 110 patients admitted to ICU during reporting were exclude from analysis. The multivariate analyses showed that an age equal to or greater than 65, and having shock and respiratory distress at the time of reporting were more likely to be admitted to the ICU. Patients who were not reported at medical centers, with bone and joint pain, and skin rash were negatively associated with ICU admission.

**Table 3:** Multivariate stepwise logistic regression analysis for ICU admission (n = 9, 087).

Variables	Univariate logistic regression analysis		Multivariate stepwise logistic regression analysis	
	OR (95% CI)	p	OR (95% CI)	p
**Age**				
<15	reference		reference	
15–39	0.09 (0.03–0.29)	0.0001	0.09 (0.03–0.27)	0.0001
40–64	0.43 (0.20–0.90)	0.026	0.39 (0.19–0.84)	0.015
65–89	6.42 (3.38–12.22)	0.0001	4.95 (2.57–9.53)	0.0001
90+	13.91 (4.4–44.00)	0.0001	9.06 (2.54–32.31)	0.001
**Gender**		0.535		0.223
Female	reference		reference	
Male	1.09 (0.84–1.42)		1.19 (0.90–1.59)	
**The levels of hospitals/clinics reporting dengue cases**				
Medical center	reference		reference	
Regional hospital	0.18 (0.13–0.23)	0.0001	0.21 (0.15–0.28)	0.0001
District hospital	0.06 (0.01–0.42)	0.005	0.093 (0.01–0.71)	0.022
Clinics	0.05 (0.02–0.11)	0.0001	0.086 (0.04–0.19)	0.0001
Other	0.07 (0.02–0.29)	0.0001	0.157 (0.04–0.69)	0.014
**Shock**	49.87 (13.30–186.95)	0.0001	8.90 (1.49–52.99)	0.016
**Respiratory distress**	19.00 (8.11–44.50)	0.0001	5.31 (1.84–15.31)	0.002
**Altered consciousness**	29.65 (6.60–133.26)	0.0001	4.79 (0.93–24.76)	0.062
**Headache**	0.39 (0.27–0.58)	0.0001	0.66 (0.44–1.00)	0.051
**Bone and joint pain**	0.31 (0.15–0.63)	0.001	0.44 (0.21–0.94)	0.034
**Skin rash**	0.18 (0.07–0.48)	0.001	0.30 (0.11–0.82)	0.019
**Abnormal liver function**	15.74 (3.04–81.57)	0.001		
**Gastrointestinal bleeding**	4.73 (1.42–15.79)	0.011		
**Bleeding**	4.51 (1.92–10.61)	0.001		
**Fatigue**	2.28 (1.43–3.65)	0.001		
**Dizziness**	2.13 (1.14–3.96)	0.017		
**Myalgia**	0.55 (0.38–0.79)	0.001		
**Fever**	0.54 (0.36–0.83)	0.005		

Variables entered on step 1 of multivariate stepwise logistic regression analysis: All variables with p ≤ 0.05 in the univariate logistic regression analysis.

Multivariate analysis showed that increasing age, respiratory distress, altered consciousness, and thrombocytopenia at the time of reporting were independent factors associated with mortality (Table 4). In contrast, myalgia, as the typical DF symptom was negatively associated with mortality. Patients who were reported at regional hospital or local clinics were negatively associated with mortality.

**Table 4:** Multivariate stepwise logistic regression analysis for mortality (N = 22, 767).

Variables	Univariate logistic regression analysis		Multivariate stepwise logistic regression analysis	
	OR (95% CI)	p	OR (95% CI)	p
**Age**	1.12 (1.10–1.13)	0.0001	1.11 (1.10–1.12)	0.0001
**Gender**		0.441		0.19
Female	reference		reference	
Male	1.12 (0.84–1.51)		1.23 (0.91–1.66)	
**The levels of hospitals/clinics reporting dengue cases**				
Medical center	reference		reference	
Regional hospital	0.61 (0.45–0.83)	0.002	0.68 (0.49–0.94)	0.018
District hospital	0.39 (0.10–1.57)	0.182	1.03 (0.24–4.46)	0.965
Clinics	0.12 (0.05–0.27)	0.0001	0.27 (0.12–0.62)	0.002
Other	0.11 (0.02–0.76)	0.025	0.33 (0.05–2.4)	0.274
**Respiratory distress**	30.12 (15.39–58.93)	0.0001	9.66 (4.57–20.44)	0.0001
**Altered consciousness**	34.40 (11.30–104.68)	0.0001	7.06 (2.19–22.73)	0.001
**Thrombocytopenia**	4.04 (2.05–7.98)	0.0001	2.55 (1.24–5.28)	0.011
**Myalgia**	0.27 (0.16–0.45)	0.0001	0.39 (0.23–0.66)	0.0001
**Bone and joint pain**	0.32 (0.14–0.73)	0.007	0.52 (0.23–1.19)	0.119
**Abnormal heart rhythm**	15.86 (1.97–127.44)	0.009		
**Shock**	15.03 (4.78–53.71)	0.0001		
**Chest tightness/ pain**	7.97 (1.89–33.49)	0.005		
**Bleeding**	3.72 (1.36–10.16)	0.010		
**Fatigue**	2.18 (1.23–3.85)	0.007		
**Fever**	0.50 (0.32–0.73)	0.002		
**Skin rash**	0.42 (0.20–0.89)	0.024		
**Headache**	0.39 (0.24–0.63)	0.0001		

Variables entered on step 1 of the multivariate stepwise logistic regression analysis: All variables with p ≤ 0.05 in the univariate logistic regression analysis.

## Discussion

To the best of our knowledge, this is the first study to employ population-based reporting data to investigate the relationship between the signs and symptoms at the time of reporting and ICU admission or mortality in Asian countries. Our study found that the corresponding rates for ICU admission and overall mortality were 3.7% and 0.8%, which were inconsistent with previous studies [1, 13, 30, 31]. In the current study, the mean age of the dengue patients with adverse prognoses was greater than 70, and the risk of ICU admission for dengue patients aged above 65 increased significantly. Of note is that for patients older than 65 or above, the risk of ICU admission was nearly 5 times greater than those less than 15 years old. Overall, the risk of mortality increased about 10% for each one-year increase in age. Multiple chronic diseases and comorbid conditions in the elderly, which are risk factors for a poor DF prognosis [31–33], may explain this phenomenon. Nonetheless, studies conducted in Singapore found that ICU admission rates were not higher in all elderly age groups [33, 34] and that only those aged between 50–59 years were at a higher risk of ICU admission [13]. Several reasons, such as sampling error, a single medical center, and acquired immunity from different dengue serotypes from prior infections in these older patients [13, 33, 34] may account for the variations in disease severity. In contrast to Singapore, no epidemic dengue serotype 2 outbreak has occurred in the last 10 years in Tainan city [4]. Therefore, most inhabitants, including the elderly citizens, were more susceptible to dengue serotype 2 infection due to a lack of protective immunity.

The study found that fever, myalgia, and headache were the most common symptoms, which is consistent with other studies from India [30], Thailand [35], Vietnam [36] and Brazil [37]. Our results also showed that the prevalence of both atypical and severe symptoms increased with age, which was in accordance with previous studies that emphasized the differences in the clinical presentations of dengue between young and elderly patients [32–34, 38]. Poor cytokine responses and more prolonged pro-inflammatory responses to various infections have been discovered in the elderly [39, 40].

The age-related changes in the adaptive immune system and the physiologic function increases the risks of various infections and leads to the presentation of atypical symptoms which may delay diagnoses in the elderly [39–41]; however, this study did not observe any delayed diagnoses for the older patients. During this most recent severe dengue outbreak in Taiwan, the level of awareness regarding dengue among healthcare providers had improved as the days between illness onset and reporting was shorter than in the previous studies in Taiwan and Singapore [23, 38]. Although the patients who died had shorter days between illness onset and dengue reporting, our analysis did not support that a delay in DF diagnosis was a major risk factor for mortality in dengue patients. Our results highlighted the rapid progress of dengue in elderly people, and found that dengue patients with adverse prognoses, the majority of whom were elderly people, presented with characteristics of severe disease, such as shock, altered consciousness, respiratory distress, and thrombocytopenia, at the time of reporting. In addition, comorbid diseases in elderly dengue patients may make adequate therapy difficult. For example, patients with DF can rapidly progress into a state of shock followed by death without prompt intravenous fluid therapy [42]. However, healthcare providers faced with a dilemma as fluid therapy must be administered cautiously to avoid cardiogenic pulmonary edemas due to the limited cardiopulmonary function commonly encountered in elderly populations [5]. The results of the current study suggest that DF diagnoses in the elderly may be challenging as the signs and symptoms can be atypical; therefore, clinicians must recognize the variations in the different presentations at the time of reporting in the different dengue patient age groups. In addition, this study also highlights the need for further research into optimal treatments for elderly dengue patients.

This study found that shock and respiratory distress as the signs and symptoms at the time of reporting were risk factors for ICU admission, which was consistent with the report from one medical center in the 2015 dengue outbreak in Tainan, Taiwan [5], and that these signs and symptoms were compatible with the warning signs or severe symptoms in the WHO 2009 classification [1]. However, the study in Singapore found that the warning signs in the WHO 2009 classification and other symptoms at first presentation were not significantly associated with ICU admissions [13]. As the study in Singapore only had a small number of ICU admissions and was conducted in only one medical center, there was possibly a low statistical power in detecting an association between the clinical symptoms and ICU admissions [13].

Our study findings were consistent with previous studies that found that altered consciousness and thrombocytopenia were risk factors for mortality in dengue patients [18, 22]. Although there were no significant associations found for bleeding signs, plasma leakage, and mortality in patients with DF in this study, thrombocytopenia has been recognized as an important factor in detecting early bleeding and plasma leakage [18], which have been identified as mortality risk factors in previous studies [18–22]. These results suggest that fatal dengue patients may progress to death rapidly before the presence of plasma leakage and major bleeding, which usually occur after day 5 of the disease [1]. This study found that dengue patients with respiratory distress were at a higher risk of mortality; however, the causes of the respiratory distress varied; sepsis, fluid overload, and upper gastrointestinal bleeding; which could have resulted in higher mortality rates without adequate management [5, 43]. The presence of respiratory distress at presentation may indicate that concurrent multi-organ damage is present and that rapid disease progress is inevitable. Taken together, a significant proportion of elderly dengue patients in the 2015 dengue outbreak presented with a fulminant course and adverse outcomes. The proportion for ICU admission and mortal DF patient were significantly higher in medical centers, indicating that the majority of critical DF patients were diagnosed and treated in the medical centers. Since national health insurance system covers nearly 99% Taiwanese people, most of the citizens can afford the medical expenses and access any level of healthcare facilities directly without economic barrier. Our findings suggested that transfer to medical centers for intensive care was inevitable for mortal or critical DF patients due to rapid progression of the disease.

There are some limitations in this study. First, this was a retrospective study and without predictive assessment that was conducted using reported data, in which the mechanisms, the risk signs, and adverse prognosis symptoms could not be fully understood as there was no information about comorbidities, primary or secondary infection status, and reasons for ICU admission or mortality in the DF patients. However, it is still worth noting that elderly DF patients with adverse prognoses presented severe and non-specific signs and symptoms at the time of reporting, and died or became critical rapidly. The current results provide a simple screen reference for the health care providers, especially the resource-limited settings, of which laboratory test is not routinely available [44]. Second, the rate of ICU admission might be underestimated due to the record of ICU admission were not documented in the early period of dengue outbreak. Third, there was a lack of standardized terminology used to describe the signs and symptoms and there was no need to report the laboratory data at the time of reporting; therefore, some of the signs and symptoms the clinical healthcare providers reported were subjective and without objective confirmation. Finally, only the signs and symptoms at the time of reporting were included for analysis, so the dynamic changes and management, which may signify additional clinical impact, were not considered. Therefore, a well-designed, prospective study is warranted that comprehensively reviews the symptoms and laboratory data for patients with acute DF.

### Conclusion

In the 2015 dengue outbreak in Tainan, patients older than 65 and those with severe and non-specific signs and symptoms at the time of reporting were at higher risk of ICU admission and mortality. Patients with adverse prognoses were of older age, had critical presentation on diagnosis, and had a rapid disease progress. First-line healthcare providers need to identify patients who are potential ICU admissions or have the possibility of dying as early as possible and be aware of atypical dengue presentations in the elderly. Moreover, preventive strategies as well as treatments specific to dengue infection in elderly people needs further study.

## Supporting information

S1 Table Categories of the signs and symptoms at the time of reporting. (PDF)
