# Supplementary material for: Symptoms associated with adverse dengue fever prognoses at the time of reporting in the 2015 dengue outbreak in Taiwan
Source: PLoS Negl Trop Dis. 2017 Dec 6;11(12):e0006091. doi: 10.1371/journal.pntd.0006091 (PMC5718413; doi:10.1371/journal.pntd.0006091)
Supplement: S1 Table — (PDF) [file pntd.0006091.s001.pdf]

S1 Table. Categories of the signs and symptoms at time of reporting.

Categories	Description of signs and symptoms by the regional medical institutions and physicians.
**Fever**	fever, fever (above 38°C), fever off and on, body temperature above 38°C
**Myalgia**	myalgia, soreness, general myalgia, general soreness, muscle ache, neck and back pain, neck pain, shoulder pain, general malaise, leg pain, low limbs pain, sore foot, foot pain, thigh pain, leg numbness, bilateral feet pain, low back pain, general soresness, backache, back pain, lumbago, back soreness, low back myalgia, flank soreness
**Headache**	headache
**Bone and joint pain**	bone pain, bone and joint pain, arthralgia, knee pain, both knees pain, bone soreness
**Skin rash**	rash, skin rash, skin lesions
**Nausea and vomiting**	nausea, vomiting, emesis, nausea and vomiting
**Gastrointestinal symptoms**	epigastria pain, epigastric pain, epigastric, lower abdominal pain, abdominal pain, abd. pain, epigastralgia, hypogastralgia, gastrectasia, AGE symptom, AGE, gastrointestinal discomfort, stomach upset, bloating, flatulence, gastric distention, abdominal fullness, abdominal dullness, epigastric dullness, diarrhea, loose stools, watery stools
**Leukopenia**	leucopenia, WBC<4000/ mm^3
**Retro-orbital pain**	retro-orbital pain, orbital pain, retroocular soreness
**Dizziness**	dizzy, lightheaded, dizziness, vertigo
**Upper respiratory symptoms**	URI, nose and throat obstruction, sneeze, rhinitis, nasal congestion, cough, cough with sputum, rhinorrhea, sore throat, sore throat, throat pain, warthm of throat
**Fatigue**	fatigue, tired, weary, malaise, weakness, muscle weakness, general weakness, quadriparesis, decreased activity
**Thrombocytopenia**	platelet≦100,000/ul, thrombocytopenia
**Poor appetite**	poor appetite, anorexia
**Chillness**	chillness, chills
**Bleeding**	bloody sputum, bleeding nose, diffuse hemorrhagic gastropathy, gingiva bleeding, petechial, bleeding gums, hematuria, blood in oral, ecchymosis
**Tourniquet test positive**	tourniquet test positive

Categories	Description of signs and symptoms by the regional medical institutions and physicians.
**Gastrointestinal bleeding**	UGI bleeding, coffee ground, LGI bleeding, black stool, tarry bloody stool
**Respiratory distress**	dyspnea, wheezing, acidosis, respiratory distress, SOB, rapid shallow breathing, short of breath
**Thirsty**	dry mouth, thirsty, dry tongue
**Itching**	itchy, itching
**Chest tightness/ pain**	chest tightness, chest discomfort, chest pain, heart burn
**Shock**	low blood pressure, hypotension, shock
**Abnormal liver function**	Hepatitis, ALT>40ku/ml, AST>40ku/ml, GOT>40ku/ml, GPT>40ku/ml
**Urinary tract injury**	acute urinary retention, frequent urination, dysuria, decreased urine output, voiding difficulties, urethritis, AKI, kidney failure, renal failure
**Altered consciousness**	altered consciousness, consciousness change, unconsciousness, confusion, confused consciousness, faint, collapse, visual hallucinations, hallucination
**Cold sweating**	cold sweating, sweats
**Accumulation of body fluids**	swelling, edema, swollen eyes, ascites, pleural effusion
**Abnormal heart rhythm**	palpitations, arrhythmia, tachycardia, bradycardia
**Epilepsy**	seizure, epilepsy
**Severe bleeding**	severe bleeding, intracranial hemorrhage, ICH
**Enlargement of lymph nodes**	enlargement of lymph nodes
**Mouth ulcers**	mouth ulcers, oral ulcer
**Hepatosplenomegaly**	hepatosplenomegaly
**Pneumonia on X-rays**	pneumonia on x-rays
**Infection**	cellulitis, infection
**Muscle symptoms**	cramp, cramping pain
